# Gastric Epithelial Neoplasms in Patients with Pulmonary Arterial Hypertension Receiving Continuous Intravenous Prostacyclin Therapy

**DOI:** 10.3390/jcm14030791

**Published:** 2025-01-25

**Authors:** Tomohiko Mannami, Takehiro Tanaka, Hiroto Shimokawahara, Kyosuke Horikawa, Yoko Shinno, Tsuyoshi Umekawa, Tsukasa Sakaki, Yasushi Fukumoto, Shin’ichi Shimizu, Isao Nozaki, Aiko Ogawa, Hiromi Matsubara

**Affiliations:** 1 Department of Gastroenterology, NHO Okayama Medical Center, Okayama 701-1192, Japan; 2 Department of Pathology and Oncology, Okayama University Graduate School of Medicine, Dentistry, and Pharmaceutical Sciences, Okayama 700-8558, Japan; 3 Department of Cardiology, NHO Okayama Medical Center, Okayama 701-1192, Japan; 4 Department of Pathology, NHO Okayama Medical Center, Okayama 701-1192, Japan; 5 Department of Surgery, NHO Okayama Medical Center, Okayama 701-1192, Japan

**Keywords:** prostacyclin, stomach, neoplasm, giant fold gastritis, foveolar-type adenoma, foveolar hyperplasia, gastric-type, gastric phenotype, prostaglandin I_2_

## Abstract

**Background**: The association of intravenous prostacyclin therapy, essential for improving prognosis and survival in pulmonary arterial hypertension (PAH), with gastric epithelial neoplasms is uncertain. This study aimed to analyze the clinicopathologic features of gastric neoplasms in patients with PAH undergoing continuous intravenous prostacyclin therapy. **Methods**: We screened the registry of patients with pulmonary hypertension who visited the NHO Okayama Medical Center. Of the patients with PAH managed between January 2003 and December 2022, those who underwent esophagogastroduodenoscopy (EGD) were assessed for gastric neoplasms. Their clinical, endoscopic, and histopathological data were reviewed. **Results**: Among the 186 patients with PAH, 56 underwent EGD, revealing 4 patients (aged 37–50 years) with gastric epithelial neoplastic lesions. All four patients received continuous intravenous prostacyclin therapy for a median of 151 months. Of the 98 patients who received prostacyclin, 28 patients underwent EGD; the incidence of gastric epithelial neoplasms was 4.1% (4/98) and the endoscopic detection rate was 14.3% (4/28). All patients had multiple tumors against a background of hypertrophic gastropathy (histologically being foveolar epithelial hyperplasia), with shared features of distal location, elevated morphology, and absent submucosal invasion. However, lymph node metastasis was observed in one lesion. By immunohistochemistry, the tumors exhibited gastric-predominant mucus phenotype and were managed by surgical or endoscopic resection without recurrence. **Conclusions**: The consistent clinicopathologic features of these cases suggest an association between continuous intravenous prostacyclin therapy and the development of hypertrophic gastropathy with potential progression to gastric epithelial neoplasia. Further prospective clinical trials are warranted to ensure safer prostacyclin use.

## 1. Introduction

Pulmonary hypertension (PH) is a pathophysiological disorder characterized by elevated pulmonary artery pressure (mean pulmonary artery pressure of ≥20 mmHg at rest) and associated with various cardiovascular and respiratory diseases [1]. The World Health Organization (WHO) classifies PH into five etiologic groups, with pulmonary arterial hypertension (PAH) categorized into WHO group 1 [2,3]. Characterized by proliferative vasculopathy exhibiting vasoconstriction, cell proliferation, fibrosis, and microthrombosis, PAH is a progressive and potentially lethal condition that ultimately leads to right heart failure and premature death [4,5,6,7]. However, the prognosis of PAH has improved with the advent of disease-specific therapies, such as prostacyclin analogs and prostacyclin receptor agonists, endothelin receptor antagonists, and nitric oxide–cyclic guanosine monophosphate enhancers. Notably, the continuous intravenous infusion of epoprostenol, a prostacyclin analog, has emerged as a cornerstone therapy for PAH, significantly improving prognosis. This therapy has resulted in improvements in symptoms, exercise capacity, hemodynamics, and increased survival rates [8,9,10] and has become the gold standard of care for high-risk patients [1,11,12].

Prostacyclin is frequently associated with gastrointestinal adverse events, including diarrhea, nausea, and vomiting, which occur in 66% to 100% of the patients with PAH [8,10,13]. These effects are generally minor and dose-dependent, and can often be managed with dose adjustments. Nonetheless, the existence of long-term and irreversible adverse events remains unclear. Recently, hypertrophic gastropathy, also referred to as giant fold gastritis, has been reported to occur in patients with PAH who receive intravenous prostacyclin infusion treatment [14]. However, the association of intravenous prostacyclin therapy with gastrointestinal neoplastic lesions has not yet been reported.

Malignant diseases contribute to the development of PH through specific mechanisms. Pulmonary tumor thrombotic microangiopathy (PTTM), a rare, yet significant, complication of malignancies, is frequently linked to gastric cancer [15,16]. Myeloproliferative neoplasms, which are malignant hematologic disorders characterized by clonal hematopoietic cell expansion, are associated with PH in 3–7% of the patients and partially account for the high cardiovascular mortality in these patients [17,18]. These observations underscore the significant cardiovascular risks faced by patients with malignancies in the setting of PH.

We recently encountered a patient with PAH who developed multiple gastric tumors during long-term intravenous prostacyclin therapy, which prompted us to conduct a retrospective review to investigate gastric epithelial neoplasms in patients with PAH. This case series details the clinicopathologic characteristics and outcomes of the gastric neoplasms identified in these patients.

## 2. Materials and Methods

**Figure 1:**
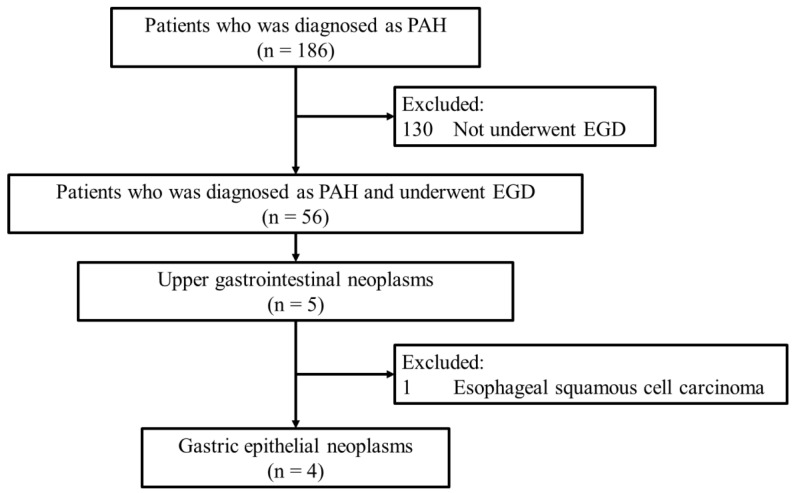
Flow chart of screening in patients with PAH and gastric epithelial neoplasia. PAH, pulmonary arterial hypertension; EGD, esophagogastroduodenoscopy.

We screened the prospectively maintained registry of patients with PH who visited the National Hospital Organization (NHO) Okayama Medical Center. Patients registered between January 2003 and December 2022 who received a diagnosis of PAH, which was classified as WHO group 1 PH based on the WHO classification system and confirmed through standard clinical and hemodynamic assessments were eligible. Additionally, patients were required to have undergone esophagogastroduodenoscopy (EGD) during the study period, with gastric epithelial neoplasms identified during the procedure. Patients with incomplete clinical records or insufficient follow-up data, and those with neoplasms located outside the upper digestive tract were excluded. A study flowchart detailing the inclusion and exclusion criteria is presented in Figure 1.

Each patient’s electronic medical record was reviewed to determine their past medical history, endoscopic findings, treatment, histopathology, and follow-up. The details of PAH treatment by cardiologists have been previously described [12]. Patients who developed neoplastic lesions were managed by a multidisciplinary team, including cardiologists, pulmonologists, gastroenterologists, anesthesiologists, and surgeons, in the NHO Okayama Medical Center. The management and treatment of gastric epithelial neoplasms adhered to the Japanese Gastric Cancer Treatment Guidelines 2021 (6th edition) [19]. Endoscopic examinations for detailed observation were conducted by board-certified endoscopists utilizing high-resolution endoscopy systems equipped with narrow-band imaging (NBI) technology (GIF-H260Z and GIF-H290Z; Olympus Medical Systems, Tokyo, Japan). Therapeutic procedures, including endoscopic mucosal resection (EMR) and endoscopic submucosal dissection (ESD), were also performed by board-certified endoscopists, following established protocols [20,21].

The histopathologic diagnosis was established through consensus among three board-certified pathologists, including one specialized in gastroenterology. The mucus phenotype of each tumor was evaluated using immunohistochemical staining for gastric markers (MUC5AC and MUC6) and intestinal markers (MUC2, CD10, and CDX2) [22]. Tumors were classified as complete intestinal, mixed (intestinal and gastric), or complete gastric phenotype, based on the degree of positivity for specific markers.

The *Helicobacter pylori* infection status was determined using one or more of the following routine diagnostic methods used in clinical practice: urea breath tests, rapid urease tests, microscopic examination and culture of biopsied specimens, stool antigen tests, and serum or urine antibody tests. Interviews were conducted to obtain detailed medical histories on past infection and eradication treatments. Patients were then categorized into three groups: uninfected (no evidence or history of *H. pylori* infection), positive (currently infected with *H. pylori*), and previously infected (cleared *H. pylori* infection following eradication therapy or spontaneous loss due to advanced atrophy).

Population-based data were obtained from the 2018 Japanese National Cancer Registry [23]. Specifically, the incidence rates of gastric cancer were calculated using age-specific population counts and age-specific gastric cancer case numbers for 2018 in Japan. These rates were then compared with the incidence rate of the study population using the chi-squared test to assess statistical significance. All statistical analyses were conducted using R version 3.6.1 (The R Foundation for Statistical Computing, Vienna, Austria).

This study was approved by the Institutional Review Board of Okayama Medical Center (2023-053) and conducted in accordance with the principles of the Declaration of Helsinki and its later amendments. All images were obtained from clinical evaluations conducted in NHO Okayama Medical Center, following the Institutional Review Board approval.

## 3. Results

Among the 186 patients with PAH identified from the PH registry, 56 patients underwent EGD, and 4 patients had epithelial neoplastic lesions in the stomach (Figure 1). The demographics and subsequent management details of the patients are outlined in Table 1. The four patients were Japanese, and their ages ranged from 37 to 50 years. They were all receiving continuous intravenous prostacyclin for 143–202 (median 151) months. None of the four patients with gastric epithelial neoplastic lesions had current *H. pylori* infection.

In the study population, 98 of the 186 patients received continuous intravenous prostacyclin therapy. The incidence of gastric epithelial neoplasms was 4.1% (4/98), which was significantly higher than the population-based incidence in age-matched Japanese individuals, which was approximately 0.01% (2574/18.8 million) (*p* < 0.001). Additionally, 28 of the 98 patients receiving continuous intravenous prostacyclin underwent EGD; therefore, the endoscopic detection rate of gastric epithelial neoplasms was 14.3% (4/28) in this subgroup of patients.

All four patients had multiple gastric epithelial tumors, ranging from two to seven in number, in a background of hypertrophic gastropathy (histologically being foveolar epithelial hyperplasia); however, no tumor-related deaths were observed. The different features of the tumors from the four patients are summarized in Table 2. All were elevated tumors, predominantly located in the distal two-thirds of the stomach, and all were resected surgically or endoscopically with no recurrence. They varied in size with no submucosal or lymphovascular invasion. However, the largest lesion (16 cm) had lymph node metastases. Most tumors showed a gastric-predominant mucus phenotype (Table 2 and Table 3).

## 4. Case Presentations

### 4.1. Case 1

**Figure 2:**
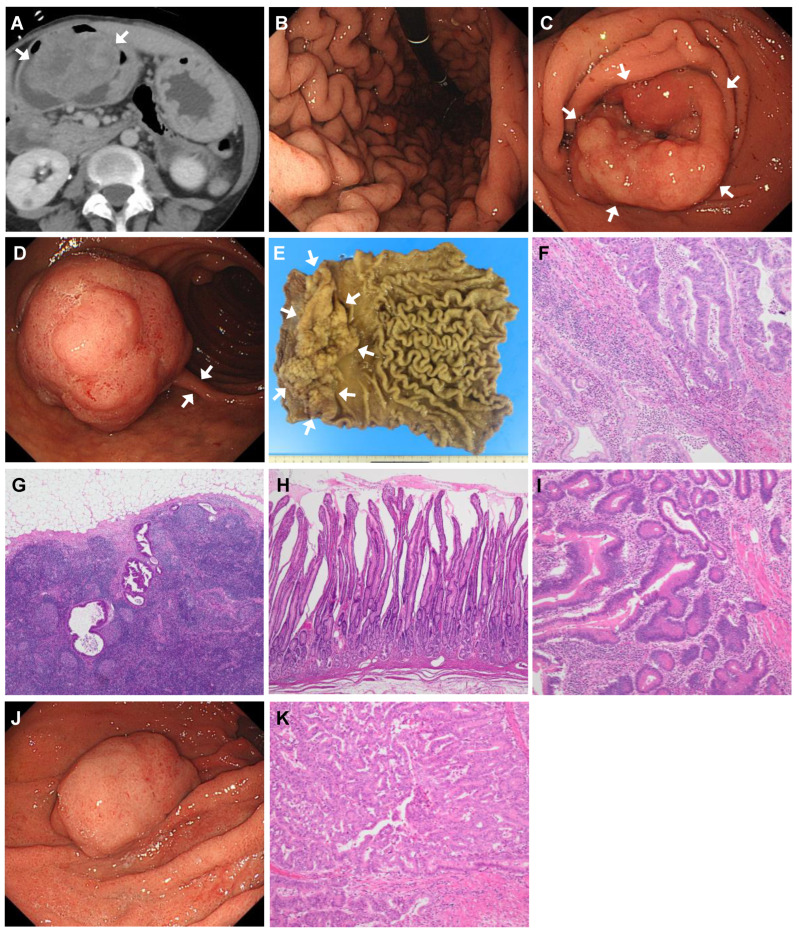
Radiologic, endoscopic, and histologic findings of Case 1. (**A**) Computed tomography image showing a tumor measuring approximately 8 cm in diameter in the distal portion of the stomach (arrows). (**B**) By upper endoscopy, the gastric mucosa is diffusely edematous and erythematous, with a granular surface pattern. The folds are markedly thickened and tortuous. (**C**) The circumferential elevated lesion in the prepyloric region (arrows). (**D**) A slightly lumpy, globular lesion is seen in the proximal portion of the descending duodenum. The lesion, approximately 3.5 cm in size, is attached to the duodenal wall by a thin stalk (arrows). (**E**) Macroscopic view of the specimen resected by distal partial gastrectomy. The prepyloric lesion is 16 cm × 9 cm in size and 4 cm in height, with a small portion of the lesion extending into the duodenal bulb (arrows). The folds of the corpus are thickened and tortuous. (**F**) Hematoxylin and eosin (H&E) staining of the prepyloric lesion showing the proliferation of tumor cells exhibiting varying degrees of dysplasia, ranging from mild to severe. (**G**) Metastatic adenocarcinoma foci are seen in a lymph node of the right greater curvature. (**H**) The marked hyperplasia of the foveolar epithelium arranged in a papillary or villous fashion, with atrophy of the pyloric glands at its depths, in the background gastric mucosa. (**I**) H&E staining of the duodenal lesion showing variably shaped tubules with complex architecture, which are composed of cells with high-grade dysplasia. The diagnosis is high-grade dysplasia. (**J**) Upper endoscopy performed 26 months after the first surgery showing a broad-based protruding lesion measuring 4 cm in the greater curvature of the remnant stomach. The lesion was not present during the previous surgery. (**K**) H&E staining of the resected lesion showing scattered areas of densely proliferating small glands with structural atypia, including cribriform structures, leading to the diagnosis of high-grade dysplasia. Slight infiltration of tumor cells into the muscularis mucosae in adjacent areas is also observed.

A 44-year-old woman was referred and admitted to the hospital with a suspected gastroduodenal mass in a CT scan she underwent to investigate right quadrant pain (Figure 2A). She was diagnosed with PAH at the age of 27 and started on continuous intravenous epoprostenol. Her brother was also diagnosed with PAH before he died. EGD revealed a diffusely edematous and erythematous gastric mucosa with markedly thickened folds (Figure 2B). A circumferential elevated mass in the prepyloric region was also detected on EGD, with a small portion of the mass extending into the duodenal bulb (Figure 2C). In addition, a pedunculated lesion measuring 3.5 cm in diameter was detected in the proximal portion of the descending duodenum (Figure 2D). Both the prepyloric and the descending duodenal lesions were simultaneously resected at a later date—the former by distal gastrectomy and the latter by ESD. Histopathological examinations of the prepyloric lesion identified intramucosal carcinoma, with a lesion measuring 16 cm × 9 cm, showing no lymphovascular invasion and no invasion into the muscularis mucosa or deeper layers (Figure 2E,F). However, a metastasis was detected at one site of the right greater curvature lymph nodes (Figure 2G). The foveolar epithelium of the nonneoplastic gastric mucosa was highly elongated with atrophic pyloric glands and marked thickening of the lamina propria mucosae measuring up to 2 cm overall (Figure 2H). High-grade dysplasia was observed on histopathology of the descending duodenal lesion (Figure 2I). A colonoscopy revealed an adenoma measuring less than 1 cm in the rectum; a wireless capsule endoscopy did not reveal any suspected lesions in the jejunum or ileum. Twenty-six months later, a surveillance EGD was performed. It revealed a newly developed semi-pedunculated 4 cm elevated lesion in the greater curvature of the remnant stomach (Figure 2J). Local excision was used to surgically resect the lesion, which was identified as high-grade dysplasia with minimal invasion to the muscularis mucosae (Figure 2K). The clinical course was favorable and there was no recurrence of endoscopy 52 months after the first surgery.

### 4.2. Case 2

**Figure 3:**
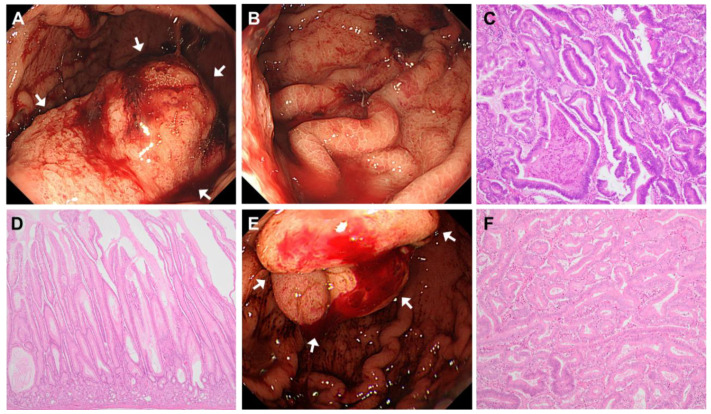
Endoscopic and histologic findings of Case 2. (**A**) Emergency endoscopy revealing a broad-based protruding lesion in the lower part of the gastric corpus exhibiting fresh bleeding (arrows). (**B**) Thickened gastric folds with diffusely edematous, erythematous mucosa and mosaic pattern are seen. (**C**) H&E staining of the lesion resected by local gastrectomy showing the presence of aberrant glandular structures with intricate branching patterns, leading to the diagnosis of high-grade dysplasia. (**D**) The background mucosa is thickened up to 2 mm, and the glandular epithelium is exhibiting marked villous and serrated overgrowth. (**E**) Upper endoscopy five months later showing a 3 cm semi-pedunculated polypoid lesion in the anterior wall of the antrum with bright red exudate (arrows). (**F**) H&E staining of the resected specimen showing tall columnar epithelial cells with increased chromatin and eosinophilic cytoplasm, forming densely proliferative papillary and tubular structures. The diagnosis is high-grade dysplasia.

A 46-year-old woman with syncope and marked anemia (hemoglobin 5.5 g/dL) was referred and admitted to the hospital for the management of bleeding from a gastric mass that was found by EGD. She was diagnosed with PAH 16 years ago and has been on continuous intravenous epoprostenol therapy for 12 years since then, followed by a continuous subcutaneous injection of treprostinil, another prostacyclin analog that has a longer half-life than epoprostenol, for the past four years. Her sister was also diagnosed with PAH before she died. An emergency endoscopy revealed that the source of the bleeding was an 8 cm mass in the lower part of the gastric corpus with bright red blood oozing from it (Figure 3A,B). Endoscopic hemostasis at the previous hospital did not work, and sustained hemostasis was not considered possible with endoscopic procedures. Therefore, the mass was surgically removed by local gastrectomy on the day of admission. Histopathological examinations revealed high-grade dysplasia with no invasion to the muscularis mucosae or deeper invasion (Figure 3C). Marked glandular hyperplasia was exhibited in the background nonneoplastic mucosa (Figure 3D). The patient was discharged on postoperative day (POD) 33 with no further progression of anemia. Five months later, she was hospitalized for renal insufficiency and pulmonary congestion. EGD performed due to worsening anemia revealed an elevated lesion measuring about 3 cm with bright red blood oozing from the antrum, which was also seen on the aforementioned endoscopy but without the bleeding (Figure 3E). Since the patient required repeated blood transfusions despite multiple endoscopic hemostasis, it was determined that bleeding control was necessary to improve her general condition, and resection of the mass by a distal partial gastrectomy was performed. Histopathology revealed high-grade dysplasia (Figure 3F). The patient developed sepsis from POD 15 through urinary tract infection and died on POD 37. An autopsy revealed that the remnant stomach showed grossly thickened walls with markedly enlarged folds, and histologically, there was diffuse foveolar epithelial hyperplasia with fundic gland dilatation. There were no residual tumors. The small and large intestine had no neoplastic lesions.

### 4.3. Case 3

**Figure 4:**
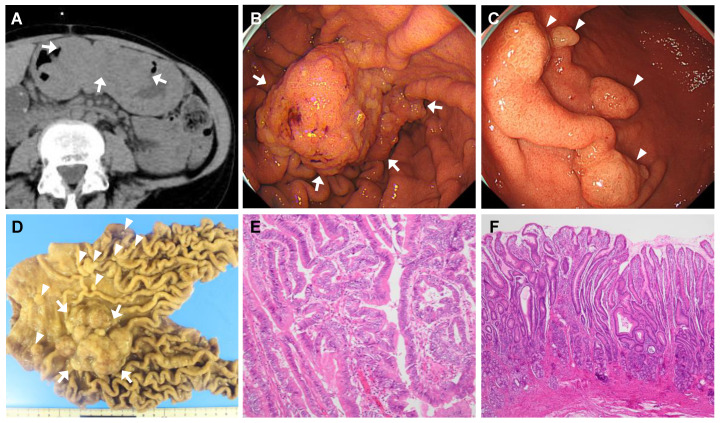
Radiologic, endoscopic, and histologic findings of Case 3. (**A**) Computed tomography image showing a gastric mass in the middle part of the stomach (arrows). (**B**) Upper endoscopy showing a huge, broad-based, protruding lesion with a nodular surface in the lower part of the gastric corpus (arrows) with thickened and snaking folds. (**C**) In addition to the main lesion, over 20 polypoid lesions are found extending from the corpus to the antrum (arrowheads). (**D**) Macroscopic findings of the specimen resected by distal gastrectomy. The main lesion located in the lesser curvature is 9 cm × 7 cm in size (arrows). Smaller polyps, up to 10 mm in size, are also seen (arrowheads). The folds of the corpus are thickened and tortuous, with some extending into the antral region. (**E**) H&E staining of the main lesion showing prominent nuclear atypia with pleomorphism and a more complex tubular architecture. (**F**) Background mucosa showing marked foveolar hyperplasia.

A 50-year-old woman diagnosed with PAH 13 years ago and placed on continuous intravenous epoprostenol one year later was admitted to the hospital because of a suspected gastric mass on a scheduled CT scan for lung field evaluation (Figure 4A). EGD revealed a large, elevated lesion with a length of nearly 10 cm from the lower part of the gastric corpus to the antrum, which was suspected to be an epithelial neoplastic lesion (Figure 4B). In addition, more than 20 polypoid lesions were located from the corpus to the antrum (Figure 4C). Based on the findings of magnifying endoscopy with NBI along with biopsy results from several lesions, the polypoid lesions in the corpus were suspected to be benign. Therefore, 16 polypoid lesions present in the corpus were first excised by EMR, all of which were histologically confirmed to be fundic gland polyps. Subsequently, the main lesion was resected by distal partial gastrectomy. Histopathological examinations revealed that the main lesion was a 9 cm × 7 cm high-grade dysplasia (Figure 4D,E). There was no invasion of the muscularis mucosa or deeper tissues. In addition to the main lesion, more than 10 polypoid lesions measuring up to 1 cm were found in the resected specimen of the antral region, six of which were low-grade dysplasia. The adjacent nonneoplastic mucosa demonstrated marked foveolar epithelial hyperplasia (Figure 4F). The patient had a good course, and no recurrence was seen on an endoscopy 23 months after surgery.

### 4.4. Case 4

**Figure 5:**
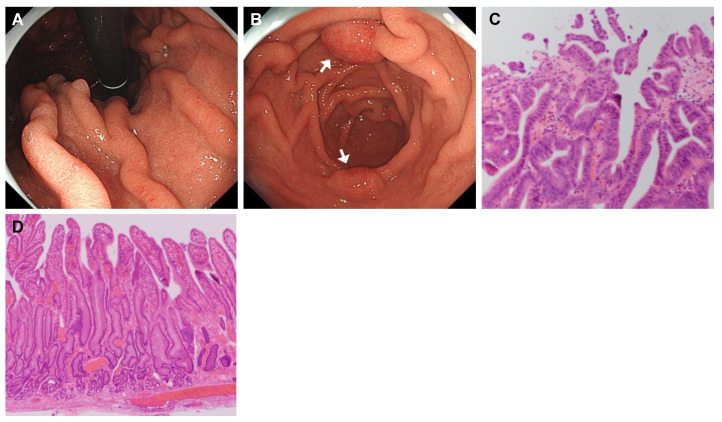
Endoscopic and histological findings of Case 4. (**A**) Upper endoscopy shows thickened giant folds throughout the stomach, not only in the greater curvature but also in the lesser curvature of the corpus. The gastric mucosa is diffusely edematous and erythematous, with a granular surface. (**B**) Two semi-pedunculated elevated lesions, approximately 2 cm in size, are located in the greater and lesser curvature of the antrum (arrows). The folds of the gastric corpus are extending into the antral region. (**C**) H&E staining of the lesion in the greater curvature showing the proliferation of atypical glands with irregular tubular structures and irregular/oval-shaped, enlarged nuclei. The final diagnosis is high-grade dysplasia. The lesion in the lesser curvature exhibits nearly identical histologic findings. (**D**) The non-neoplastic mucosa adjacent to the tumors exhibits foveolar hyperplasia and atrophy of the deeper pyloric glands.

A 37-year-old man was admitted to the hospital for the management of gastric neoplastic lesions, which were found during an annual medical checkup. He was diagnosed with idiopathic PAH 17 years earlier and had been on continuous intravenous infusions of epoprostenol for 12 years. EGD revealed giant folds from the corpus to the antrum of the stomach, and two semi-pedunculated elevated lesions were located at the antral region, both of which measured approximately 2 cm in diameter (Figure 5A,B). Biopsies of these two lesions were suspicious of high-grade dysplasia. Both tumors were curatively resected by ESD and had similar histological findings, with the final diagnosis of high-grade dysplasia (Figure 5C). Foveolar epithelial hyperplasia was observed in the nonneoplastic gastric mucosa surrounding the lesions (Figure 5D). The patient was discharged on POD 12 with a good postoperative course, and an endoscopy performed 17 months after the resection revealed no recurrence.

## 5. Discussion

In this study, we presented four cases of gastric epithelial neoplasm that occurred in patients with PAH. These cases had the following features in common. The affected patients were relatively young and treated with continuous intravenous prostacyclin therapy; their stomachs had giant folds with histologic findings being foveolar epithelial hyperplasia.

The first important finding is that all four patients with PAH who developed gastric epithelial neoplasms were relatively young (aged 37–50 years), and they were on continuous intravenous prostacyclin therapy. Gastric cancer is the fifth leading cause of cancer-related death. It is relatively common worldwide, with an estimated 968,784 new cases and 660,175 deaths recorded in 2022 [24]. However, it usually affects elderly patients with an average age at diagnosis of 60 years for early-stage gastric cancer and approximately 70 years for advanced gastric cancer, with similar trends in both Asian and Western countries [25,26]. However, the patients included in this case series were significantly younger, and they were predominantly women, perhaps because PAH predominantly develops in women in their 30s and 40s.

Additionally, the affected patients were only those on continuous intravenous prostacyclin therapy. The incidence of gastric epithelial neoplasms in this population was 4.1% (4/98), which was significantly higher than the population-based incidence in Japanese individuals. Moreover, the endoscopic detection rate of gastric epithelial neoplasms among patients receiving continuous intravenous prostacyclin therapy was 14.3% (4/28), indicating that gastric epithelial neoplasia was detected by EGD in approximately one in seven patients on continuous intravenous prostacyclin therapy. It is plausible that more frequently performed endoscopic examinations could identify additional patients, potentially increasing the rate. Although rational explanations for this high occurrence rate are elusive, given that none of the patients who did not receive continuous intravenous prostacyclin therapy developed the disease, it suggests that prostacyclin use might increase the incidence of gastric epithelial neoplasia. This first case series of gastric epithelial neoplasms in four of our patients with PAH provides a new perspective on the need to monitor gastric tumors while managing PAH cases, even in young adult patients.

The second important observation in our study is that all four cases in which gastric epithelial neoplasia occurred had gastric giant folds. The histology of the gastric mucosa in these cases exhibited foveolar epithelial hyperplasia. This finding is consistent with cases described in two previously published studies. One of these cases was that of a 48-year-old man who had been receiving continuous infusions of prostacyclin for five years after being diagnosed with IPAH. Endoscopy revealed enlarged gastric folds, and biopsies with histopathology revealed foveolar epithelial hyperplasia [27]. Recently, a study was conducted in which gastric barium imaging was used to assess the prevalence of hypertrophic gastropathy in twelve patients with PAH who were treated with prostacyclin and four patients who did not use prostacyclin. They found that giant gastric folds were significantly more common in patients treated with prostacyclin (75% vs. 0%; *p* = 0.019) [14]. The authors hypothesized that prostacyclin could be associated with hypertrophic gastropathy. The present report further supports the implication that long-term prostacyclin therapy might be associated with hypertrophic gastropathy, which could warrant referring to this condition as prostacyclin-associated gastropathy.

The association between the use of prostacyclin for PAH, hypertrophic gastropathy, and gastric epithelial neoplasia in these cases is of significant interest. One potential contributing factor is that gastric neoplasms might have triggered the onset of PH. PTTM can lead to PH through intraluminal obstruction by tumor cells, fibrin deposition, and intimal fibrocellular proliferation [16]. PTTM has been reported in 3.3% of autopsied patients with malignancy and in up to 16% of patients with gastric cancer. However, PTTM involvement is unlikely in the cases reported here. PTTM typically occurs in advanced cancer with poor prognosis, presenting as acute respiratory distress and leading to death within days [15]. Even with chemotherapy for gastric cancer, the median survival is only 9.5 weeks in these patients [16]. In contrast, the median duration from the initiation of prostacyclin therapy to tumor diagnosis was 151 months in our cases, rendering the possibility of advanced gastric cancer at the time of PAH onset improbable.

Another potential explanation for the observed association lies in the role of prostacyclin in gastric mucosal pathology. While prostacyclin plays an important role in upper GI tract mucosal protection [28,29], the mechanism by which foveolar epithelial hyperplasia occurs is unknown. Known pathological mechanisms by which foveolar epithelial hyperplasia occurs include COX-2 overexpression during hypergastrinemia and increased signaling of the epidermal growth factor receptor driven by the overproduction of transforming growth factor-alpha [27,30,31]. However, it is unclear whether prostacyclin could induce these mechanisms. In our case series, it should be noted that prostacyclin administration was performed at rather high doses and for long periods. In the aforementioned clinical study showing significantly more hypertrophic gastropathy in the prostacyclin use group (in which no gastric neoplasms occurred), the median dose of epoprostenol was 38 ng/kg/min (interquartile range [IQR] 32.5–52), and the median duration of use was 7 years (IQR 5–9) [14]. In our four cases in which gastric neoplasms occurred, the dose of epoprostenol ranged from 72.7 to 136.8 ng/kg/min, and the duration of use ranged from 12 to 16 years. Although whether or not this difference in dose and duration is related to carcinogenesis is unclear, it is intriguing that gastric epithelial neoplasms developed only in our cases with the administration of the high prostacyclin load. In addition, the highly similar clinicopathologic picture of the neoplastic lesions strongly implies that they share a common underlying cause and mechanism of tumorigenesis. Further studies are needed to determine whether a pathway exists from prostacyclin-associated gastropathy to prostacyclin-associated gastric epithelial neoplasia.

It should also be noted that there were lymph node metastases in one case. The lack of invasion into the submucosal layer, even after massive growth in three of our cases, appears to show that the local invasive potential of these lesions is not high. However, the presence of nodal involvement in Case 1, albeit regional but not a distant metastasis, indicates that these gastric neoplasms are potentially lethal and have the potential ability to progress to an advanced stage. Patients with gastric cancer diagnosed at an advanced stage have a 5-year survival rate of less than 10% [24], whereas those diagnosed at an early stage and treated endoscopically have a significantly better prognosis with a 5-year survival rate of more than 95% [32]. The early detection of gastric cancer significantly improves the prognosis of patients and allows them to preserve the organ [33], with minimally invasive interventions, which is crucial for patients with PAH who are susceptible to hemodynamic instability. Indeed, only one of the cases (Case 4), a patient with no symptoms, whose tumor was found during a routine EGD for a medical checkup, was able to receive endoscopic treatment and avoid surgical gastrectomy because the lesion was found to be much smaller than those in the other three cases. Routine surveillance endoscopy in patients on continuous intravenous prostacyclin might lead to early detection and minimally invasive interventions of gastric epithelial neoplasia.

This retrospective case series has inherent methodologic limitations not present in controlled studies. First, the absence of a control group restricts our ability to establish a causality between continuous intravenous prostacyclin therapy and the development of gastric epithelial neoplasia and the observed associations should be interpreted with caution. Second, the small sample size of four cases limits the generalizability of our findings to larger populations. Third, selection bias might have influenced the study results. All four patients included in the study were Japanese, which might restrict the applicability of our findings to populations with different genetic or environmental backgrounds. Fourth, this study did not include detailed imaging analyses. Investigating endoscopic characteristics of the gastric mucosa associated with epithelial neoplasms, computed tomographic findings of gastric epithelial neoplasms, and endoscopic features of hypertrophic gastropathy in patients receiving prostacyclin therapy can provide additional insights into this phenomenon. Further prospective studies with larger cohorts and control groups and consideration of the impact of prostacyclin dosage are essential to validate these findings and explore potential mechanisms underlying the observed associations.

## 6. Conclusions

In this first case series of gastric epithelial neoplasms observed in patients with PAH, the consistent clinicopathologic features indicate a potential association between prostacyclin use and the development of hypertrophic gastropathy, which may progress to gastric epithelial neoplasia. Physicians should be vigilant regarding gastric tumor development during the management of patients with PAH, particularly those receiving prostacyclin therapy. Further accumulation of cases and the analysis of larger cohorts are warranted to determine whether regular surveillance endoscopy should be utilized to ensure the safety of treatment with prostacyclin, an essential drug for structuring optimal treatment strategies for PAH.

## Figures and Tables

**Table 1:** Demographics and subsequent management details of the patients.

Patient	1	2	3	4
Age (year)	44	46	50	37
Sex	Female	Female	Female	Male
BMI (kg/m^2^)	16	27.8	17.5	21.1
Etiology of PAH	HPAH	HPAH	CHD-PAH (ASD)	IPAH
Duration of epoprostenol use (months)	202	143 *	149	152
Dose of epoprostenol (ng/kg/min)	72.7	136.8	103.6	97.4
Other PAH-specific drugs (dose/day)	Macitentan 10 mg Sildenafil 60 mg	Macitentan 10 mg Tadalafil 40 mg	Macitentan 10 mg Tadalafil 20 mg	None
*H. pylori* infection status	Previously infected	Uninfected	Uninfected	Previously infected
PPI use	Esomeprazole	Rabeprazole	Lansoprazole	Esomeprazole
EGD indication	Right quadrant pain	Anemia	Gastric mass on CT	Medical checkup
Giant fold	Present	Present	Present	Present
Histopathological foveolar epithelial hyperplasia at surrounding nonneoplastic lesions	Present	Present	Present	Present
Number of epithelial neoplasms of the stomach	2	2	7	2
Metachronous gastric neoplasia that occurred afterward	Present	Absent	Absent	Absent
Follow-up period (months)	56	7	30	22
Outcome	Alive	Deceased †	Alive	Alive

* Followed by continuous subcutaneous treprostinil injection for the past 40 months. † Not due to a tumor-related cause. BMI, body mass index; PAH, pulmonary arterial hypertension; HPAH, heritable pulmonary arterial hypertension; CHD, congenital heart disease; ASD, atrial septal defect; PPI, proton pump inhibitor; EGD, esophagogastroduodenoscopy; CT, computed tomography.

**Table 2:** Histopathological characteristics of gastric neoplastic lesions from the four patients.

Patient	Location	Macroscopic Type	Size (cm)	Therapy	Diagnosis	Mucus Phenotype (Predominant)	Submucosal Invasion	Lymphovascular Invasion	Lymph Node Metastasis	Endoscopic Follow-Up Period (Months)	Recurrence
1	L	Elevated	16	Surgery	Intramucosal carcinoma	Mixed (intestinal)	Absent	Absent	Present	52	Absent
	U	Elevated	4	Surgery	HGD	Gastric	Absent	Absent	Absent	23	Absent
2	M	Elevated	8	Surgery	HGD	Mixed (gastric)	Absent	Absent	Absent	6	Absent
	L	Elevated	3.5	Surgery	HGD	Mixed (gastric = intestinal)	Absent	Absent	Absent	n/a *	n/a *
3	L	Elevated	9	Surgery	HGD	Mixed (gastric)	Absent	Absent	Absent	23	Absent
	L	Elevated	1	Surgery	LGD	Mixed (gastric)	Absent	Absent	Absent	23	Absent
	L	Elevated	1	Surgery	LGD	Mixed (gastric)	Absent	Absent	Absent	23	Absent
	L	Elevated	1	Surgery	LGD	Mixed (gastric)	Absent	Absent	Absent	23	Absent
	L	Elevated	1	Surgery	LGD	Mixed (gastric)	Absent	Absent	Absent	23	Absent
	L	Elevated	1	Surgery	LGD	Mixed (gastric)	Absent	Absent	Absent	23	Absent
	L	Elevated	1	Surgery	LGD	Mixed (gastric)	Absent	Absent	Absent	23	Absent
4	L	Elevated	2	ESD	HGD	Mixed (gastric)	Absent	Absent	n/a †	17	Absent
	L	Elevated	2	ESD	HGD	Mixed (gastric)	Absent	Absent	n/a †	17	Absent

* No endoscopy was performed because the patient died after two months of treatment. † No lymph nodes were sampled because of endoscopic treatment. U, upper third of the stomach; M, middle third of the stomach; L, lower third of the stomach; HGD, high-grade dysplasia; LGD, low-grade dysplasia; ESD, endoscopic submucosal dissection; n/a, not available.

**Table 3:** Mucus phenotype and immunohistochemical staining results of gastric neoplastic lesions in four cases.

Patient	Diagnosis *	Mucus Phenotype (Predominant)	MUC5AC	MUC6	MUC2	CD10	CDX2
1	Intramucosal carcinoma	Mixed (intestinal)	p+	p+	+	p+	+
	HGD	Gastric	+	+	−	−	−
2	HGD	Mixed (gastric)	+	+	−	−	p+
	HGD	Mixed (gastric = intestinal)	p+	p+	p+	p+	+
3	HGD	Mixed (gastric)	+	−	p+	−	+
	LGD	Mixed (gastric)	+	+	−	p+	p+
	LGD	Mixed (gastric)	+	+	−	p+	p+
	LGD	Mixed (gastric)	+	−	−	−	p+
	LGD	Mixed (gastric)	+	p+	−	−	p+
	LGD	Mixed (gastric)	+	p+	−	−	p+
	LGD	Mixed (gastric)	+	+	−	−	p+
4	HGD	Mixed (gastric)	+	p+	−	p+	p+
	HGD	Mixed (gastric)	+	+	p+	p+	p+

Immunohistochemical staining positivity for each monoclonal antibody is described as follows: −, negative (<5% of neoplastic cells stained); p+, partially positive (5–60% of neoplastic cells stained); +, positive (>60% of neoplastic cells stained). * The order of each lesion within the column is consistent with Table 2; MUC5AC and MUC6 were used as gastric markers, while MUC2, CD10, and CDX2 were intestinal markers. HGD, high-grade dysplasia; LGD, low-grade dysplasia.

## Data Availability

The original contributions presented in this study are included in the article. Further inquiries can be directed to the corresponding author.

## References

[1] Humbert M., Kovacs G., Hoeper M.M., Badagliacca R., Berger R.M.F., Brida M., Carlsen J., Coats A.J.S., Escribano-Subias P., Ferrari P. (2023). 2022 ESC/ERS Guidelines for the diagnosis and treatment of pulmonary hypertension. Eur. Respir. J..

[2] Simonneau G., Montani D., Celermajer D.S., Denton C.P., Gatzoulis M.A., Krowka M., Williams P.G., Souza R. (2019). Haemodynamic definitions and updated clinical classification of pulmonary hypertension. Eur. Respir. J..

[3] Kovacs G., Bartolome S., Denton C.P., Gatzoulis M.A., Gu S., Khanna D., Badesch D., Montani D. (2024). Definition, classification and diagnosis of pulmonary hypertension. Eur. Respir. J..

[4] Rubin L.J. (1997). Primary pulmonary hypertension. N. Engl. J. Med..

[5] Farber H.W., Loscalzo J. (2004). Pulmonary arterial hypertension. N. Engl. J. Med..

[6] Chin K.M., Rubin L.J. (2008). Pulmonary arterial hypertension. J. Am. Coll. Cardiol..

[7] Adão R., Perez-Vizcaino F., Redwan B., Brás-Silva C. (2024). Editorial: Therapeutics in pulmonary arterial hypertension. Front. Cardiovasc. Med..

[8] Rubin L.J., Mendoza J., Hood M., McGoon M., Barst R., Williams W.B., Diehl J.H., Crow J., Long W. (1990). Treatment of primary pulmonary hypertension with continuous intravenous prostacyclin (epoprostenol). Results of a randomized trial. Ann. Intern. Med..

[9] Barst R.J., Rubin L.J., Long W.A., McGoon M.D., Rich S., Badesch D.B., Groves B.M., Tapson V.F., Bourge R.C., Brundage B.H. (1996). A comparison of continuous intravenous epoprostenol (prostacyclin) with conventional therapy for primary pulmonary hypertension. N. Engl. J. Med..

[10] Badesch D.B., Tapson V.F., McGoon M.D., Brundage B.H., Rubin L.J., Wigley F.M., Rich S., Barst R.J., Barrett P.S., Kral K.M. (2000). Continuous intravenous epoprostenol for pulmonary hypertension due to the scleroderma spectrum of disease. A randomized, controlled trial. Ann. Intern. Med..

[11] Jacobs W., Vonk-Noordegraaf A. (2009). Epoprostenol in pulmonary arterial hypertension. Expert Opin. Drug Metab. Toxicol..

[12] Sugiyama Y., Matsubara H., Shimokawahara H., Ogawa A. (2022). Outcome of mean pulmonary arterial pressure-based intensive treatment for patients with pulmonary arterial hypertension. J. Cardiol..

[13] Sitbon O., Delcroix M., Bergot E., Boonstra A.B., Granton J., Langleben D., Subias P.E., Galie N., Pfister T., Lemarie J.C. (2014). EPITOME-2: An open-label study assessing the transition to a new formulation of intravenous epoprostenol in patients with pulmonary arterial hypertension. Am. Heart J..

[14] Miura Y., Kataoka M., Chiba T., Inami T., Yoshino H., Satoh T. (2018). Giant Fold Gastritis Induced by Epoprostenol Infusion in Patients with Pulmonary Arterial Hypertension. Circ. J..

[15] von Herbay A., Illes A., Waldherr R., Otto H.F. (1990). Pulmonary tumor thrombotic microangiopathy with pulmonary hypertension. Cancer.

[16] Godbole R.H., Saggar R., Kamangar N. (2019). Pulmonary tumor thrombotic microangiopathy: A systematic review. Pulm. Circ..

[17] Adir Y., Humbert M. (2010). Pulmonary hypertension in patients with chronic myeloproliferative disorders. Eur. Respir. J..

[18] Todor S.B., Ichim C., Boicean A., Mihaila R.G. (2024). Cardiovascular Risk in Philadelphia-Negative Myeloproliferative Neoplasms: Mechanisms and Implications-A Narrative Review. Curr. Issues Mol. Biol..

[19] Association J.G.C. (2023). Japanese Gastric Cancer Treatment Guidelines 2021 (6th edition). Gastric Cancer.

[20] Bhatt A., Abe S., Kumaravel A., Vargo J., Saito Y. (2015). Indications and Techniques for Endoscopic Submucosal Dissection. Am. J. Gastroenterol..

[21] Hoffman A., Atreya R., Rath T., Neurath M.F. (2021). Current Endoscopic Resection Techniques for Gastrointestinal Lesions: Endoscopic Mucosal Resection, Submucosal Dissection, and Full-Thickness Resection. Visc. Med..

[22] Tsukashita S., Kushima R., Bamba M., Sugihara H., Hattori T. (2001). MUC gene expression and histogenesis of adenocarcinoma of the stomach. Int. J. Cancer.

[23] National Cancer Center, Japan Cancer Statistics in Japan. National Cancer Registry (Ministry of Health, Labour and Welfare), tabulated by Cancer Information Service. https://ganjoho.jp/reg_stat/statistics/data/dl/en.html.

[24] Ferlay J., Ervik M., Lam F., Laversanne M., Colombet M., Mery L., Piñeros M., Znaor A., Soerjomataram I., Bray F. (2024). Global Cancer Observatory: Cancer Today.

[25] Eckardt V.F., Giessler W., Kanzler G., Remmele W., Bernhard G. (1990). Clinical and morphological characteristics of early gastric cancer. A case-control study. Gastroenterology.

[26] Everett S.M., Axon A.T. (1997). Early gastric cancer in Europe. Gut.

[27] Burdick J.S., Chung E., Tanner G., Sun M., Paciga J.E., Cheng J.Q., Washington K., Goldenring J.R., Coffey R.J. (2000). Treatment of Menetrier’s disease with a monoclonal antibody against the epidermal growth factor receptor. N. Engl. J. Med..

[28] Wallace J.L., Tigley A.W. (1995). Review article: New insights into prostaglandins and mucosal defence. Aliment. Pharmacol. Ther..

[29] Amagase K., Izumi N., Takahira Y., Wada T., Takeuchi K. (2014). Importance of cyclooxygenase-1/prostacyclin in modulating gastric mucosal integrity under stress conditions. J. Gastroenterol. Hepatol..

[30] Kanda N., Seno H., Kawada M., Sawabu T., Uenoyoma Y., Nakajima T., Konda Y., Fukui H., Takeuchi T., Chiba T. (2006). Involvement of cyclooxygenase-2 in gastric mucosal hypertrophy in gastrin transgenic mice. Am. J. Physiol. Gastrointest. Liver Physiol..

[31] Almazar A.E., Penfield J.D., Saito Y.A., Talley N.J. (2021). Survival Times of Patients with Menetrier’s Disease and Risk of Gastric Cancer. Clin. Gastroenterol. Hepatol..

[32] Hasuike N., Ono H., Boku N., Mizusawa J., Takizawa K., Fukuda H., Oda I., Doyama H., Kaneko K., Hori S. (2018). A non-randomized confirmatory trial of an expanded indication for endoscopic submucosal dissection for intestinal-type gastric cancer (cT1a): The Japan Clinical Oncology Group study (JCOG0607). Gastric Cancer.

[33] Zhang X., Li M., Chen S., Hu J., Guo Q., Liu R., Zheng H., Jin Z., Yuan Y., Xi Y. (2018). Endoscopic Screening in Asian Countries Is Associated with Reduced Gastric Cancer Mortality: A Meta-analysis and Systematic Review. Gastroenterology.

